# Second primary tumor after immune checkpoint inhibitor therapy: A case report

**DOI:** 10.1111/1759-7714.14327

**Published:** 2022-02-11

**Authors:** Kang Miao, Shuangni Yu, Jun Ni, Xiaotong Zhang, Li Zhang

**Affiliations:** ^1^ Department of Pulmonary and Critical Care Medicine, Peking Union Medical College Hospital Chinese Academy of Medical Science & Peking Union Medical College Beijing China; ^2^ Department of Pathology, Peking Union Medical College Hospital Chinese Academy of Medical Science & Peking Union Medical College Beijing China

**Keywords:** case report, immune checkpoint inhibitor, non‐small cell lung cancer, second primary tumor

## Abstract

Immune checkpoint inhibitors (ICIs) are used to treat many types of cancers. However, the effect of ICIs on second primary tumors is still unclear. Some studied have concluded that ICIs could reduce the incidence of second primary tumors, while others found an increased overall risk of second primary cancer after the introduction of ICIs to the treatment of melanoma. Here, we report the case of a patient with advanced non‐small cell lung cancer (NSCLC) who was treated with ICIs in combination with antiangiogenic drugs, and subsequently developed a second primary tumor in the context of a favorable curative effect of the primary lung cancer. From this case, we know that good efficacy of ICIs for a primary tumor does not mean that a second primary tumor will never develop, which reminds clinicians to consider the possibility of a second primary tumor rather than treating it directly as disease progression.

## Graphical abstract

Here, we report a case of advanced non‐small cell lung cancer (NSCLC) patient, who treated with ICIs in combination with anti‐angiogenic drugs, developed a second primary tumor in the context of a favorable curative effect of the primary lung cancer. From the case, we can know that good efficacy of ICIs for a primary tumor does not mean that a second primary tumor will never develop, which reminds clinicians to consider the possibility of a second primary tumor rather than treating it directly as disease progression.

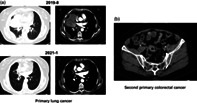

## INTRODUCTION

Immune checkpoint inhibitors (ICIs) are a class of antitumor drugs that activate the immune system to kill tumor cells by inhibiting immune checkpoints on the surface of immune or tumor cells. ICIs are used to treat many types of cancers including melanoma, lung cancer, colorectal cancer, uroepithelial cancer, liver cancer, etc.^1^ Different types of tumors respond differently to ICIs. In addition to the progression of the primary tumor, the development of a second primary tumor also has an adverse effect on a patient's prognosis.^2^ However, the effect of ICIs on a second primary tumor is still unclear. Here, we report the case of an advanced non‐small cell lung cancer (NSCLC) patient who was treated with ICIs in combination with antivascular therapy, and developed a second primary tumor in the context of a favorable curative effect of the primary lung cancer.

## CASE REPORT

The patient in this study was a 66‐year‐old female, diagnosed with right lung adenocarcinoma (cT4N3M1c, stage IVb) with brain metastases, liver metastases and multiple abdominal lymph node metastases in October 2017. Next‐generation sequencing (NGS) of punctured tissue biopsy suggested ERBB2 p.Q78L (+), KRAS p.G12V (+), TP53 p.R342X (+), tumor mutational burden (TMB) 16.52 Muts/Mb, microsatellite stability (MSS); PD‐L1 expression (−). From October 2017 to July 2019, she received five lines of treatment: first‐line treatment was with pemetrexed combined with carboplatin followed by pemetrexed monotherapy maintenance (October 2017–July 2018); second‐line treatment was with docetaxel combined with bevacizumab (August 2018–November 2018); third‐line treatment was with gemcitabine monotherapy (December 2018–February 2019); fourth‐line treatment was with anlotinib hydrochloride combined with afatinib (March 2019–May 2019); and fifth‐line treatment was with vincristine combined with bevacizumab (June 2019–July 2019).

In August 2019, the disease was again found to have progressed. Although the patient's PD‐L1 expression was negative, considering that chemotherapy could no longer control tumor progression while ICIs had not previously been administered, the sixth‐line regimen of ICIs combined with antiangiogenic therapy: camrelizumab 200 mg q. 2 weeks + apatinib 250 mg q.d. was chosen. After two cycles of treatment, the patient's general condition improved significantly and the imaging efficacy assessment confirmed a partial response (PR). The efficacy continued to maintain PR, and carcinoembryonic antigen (CEA) was gradually decreased from 9.4 ug/l to 3.0 ug/l until December 2020. No significant adverse effects were observed during drug administration, except for mild reactive cutaneous capillary endothelial proliferation (RCCEP) and elevated blood pressure.

**FIGURE 1:**
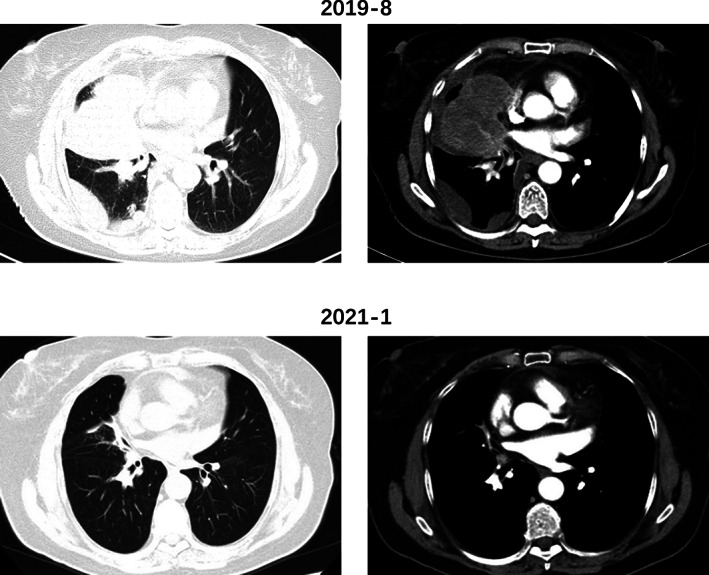
Chest CT before and after treatment with camrelizumab + apatinib

**FIGURE 2:**
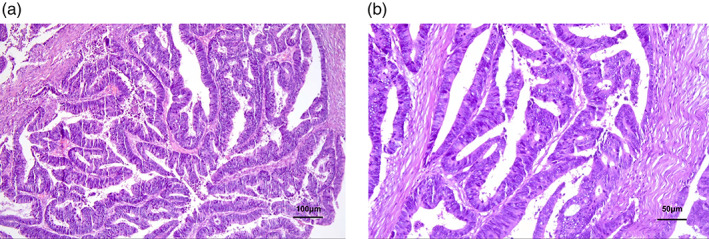
Pathology of colorectal cancer

At the end of 2020, the frequency of diarrhea had increased from once a day to 3–4 times/day, and the fecal characteristics changed from yellow formed soft stools to unformed loose stools. In addition, the CEA gradually increased to 8.0 ug/l. Chest computed tomography (CT) in January 2021 showed that the original tumor in the middle lobe of the right lung had almost completely disappeared with only a few cords remaining, and the multiple lymph nodes in both axillae, mediastinum and hilum were significantly smaller than before. The efficacy evaluation of the primary lesion remained significant PR (Figure 1). However, abdominal CT suggested a thickening of the intestinal wall at the junction of the rectum and sigmoid colon (Figure S1B). Colonoscopy revealed an ulcerated bulging mass in the rectosigmoid junction, and the biopsy pathology showed high‐grade intraepithelial neoplasia, which was considered as a second primary cancer. Thus, camrelizumab and apatinib therapy was suspended, and the patient underwent radical surgery for rectosigmoid junction cancer in March 2021. Postoperative pathology showed a moderately differentiated adenocarcinoma of the colon, invading the deep muscular layer of the intestinal wall without invading the plasma membrane, while no metastasis was seen in any of the lymph nodes (Figure 2). Immunohistochemistry showed MLH‐1(+), MSH‐2(+), MSH‐6(+), PMS‐2(+), suggesting normal DNA mismatch repair (MMR) function. Two months after surgery, chest CT revealed a slightly enlarged intrapulmonary lesion, thus treatment with camrelizumab and apatinib was restarted in May 2021 and the primary lung cancer has continued to remain stable to date.

## DISCUSSION

The response of different tumor types to ICIs is not entirely consistent. Colorectal and lung cancer are both types of disease that may benefit from ICI therapy, yet differences exist in the predictors of their efficacy. For non‐small cell lung cancer, PD‐L1 expression and TMB may better reflect the efficacy of ICIs,^3^ while for colorectal cancer, microsatellite instability‐high (MSI‐H) or deficient mismatch repair (dMMR) may better reflect the prognosis after ICI treatment.^4^ In our case, the patient presented with a new tumor in the colorectal region in the context of a favorable curative effect of primary lung cancer. The surgical specimen was pathologically confirmed as a primary adenocarcinoma of the colon with intermediate differentiation. As for lung cancer, the patient had a high TMB load despite being PD‐L1 expression negative, suggesting a high likelihood that she would benefit from ICIs. The results showed that camrelizumab in combination with apatinib brought significant PR and at least 17 months progression‐free survival (PFS) for this patient. For colorectal cancer, immunohistochemistry suggested normal DNA mismatch repair function, consistent with the prediction of no significant effect of colorectal cancer with ICIs.

The development of a second primary tumor is one of the important risk factors for poor survival prognosis of patients who have had a satisfactory therapeutic effect of their primary tumor. More than 5% of patients will develop a second primary tumor after the diagnosis of a primary tumor.^5^ However, the effect of ICIs on second primary tumors is not yet clear. Heudel et al. concluded that ICIs could reduce the incidence of second primary tumors,^6^, ^7^ while Deng et al. found an increased overall risk of second primary cancer after the introduction of ICIs to the treatment of melanoma.^8^ In theory, the immune effect activated by ICIs is systemic and should be able to produce an offensive effect against all types of tumors. However, patients who benefit from ICIs tend to have higher TMB, implying a higher frequency of genetic mutations than normal.^9^ Thus, the increased survival time that immunotherapy brings to tumor patients also increases the probability of a second primary tumor caused by a newly generated gene mutation.

In clinical practice, when a de novo tumor develops during treatment with ICIs, the first consideration would be tumor metastasis and the choice of subsequent treatment regimen would be completely different. We were unable to make a definitely direct association between the development of colorectal cancer and usage of ICIs. However, we know that good efficacy of ICIs for a primary tumor does not mean that a second primary tumor will never develop, which reminds clinicians to consider the possibility of a second primary tumor rather than treating it directly as disease progression.

## CONFLICT OF INTEREST

The authors have no conflicts of interest to declare.

## Supporting information


**Fig. S1** (A) Pathology of lung cancer. (B) Abdominal CT of colorectal cancer.
