# Supplementary figures and images for: Second primary tumor after immune checkpoint inhibitor therapy: A case report
Source: Thorac Cancer. 2022 Feb 11;13(7):1076–8. doi: 10.1111/1759-7714.14327 (PMC8977149; doi:10.1111/1759-7714.14327)

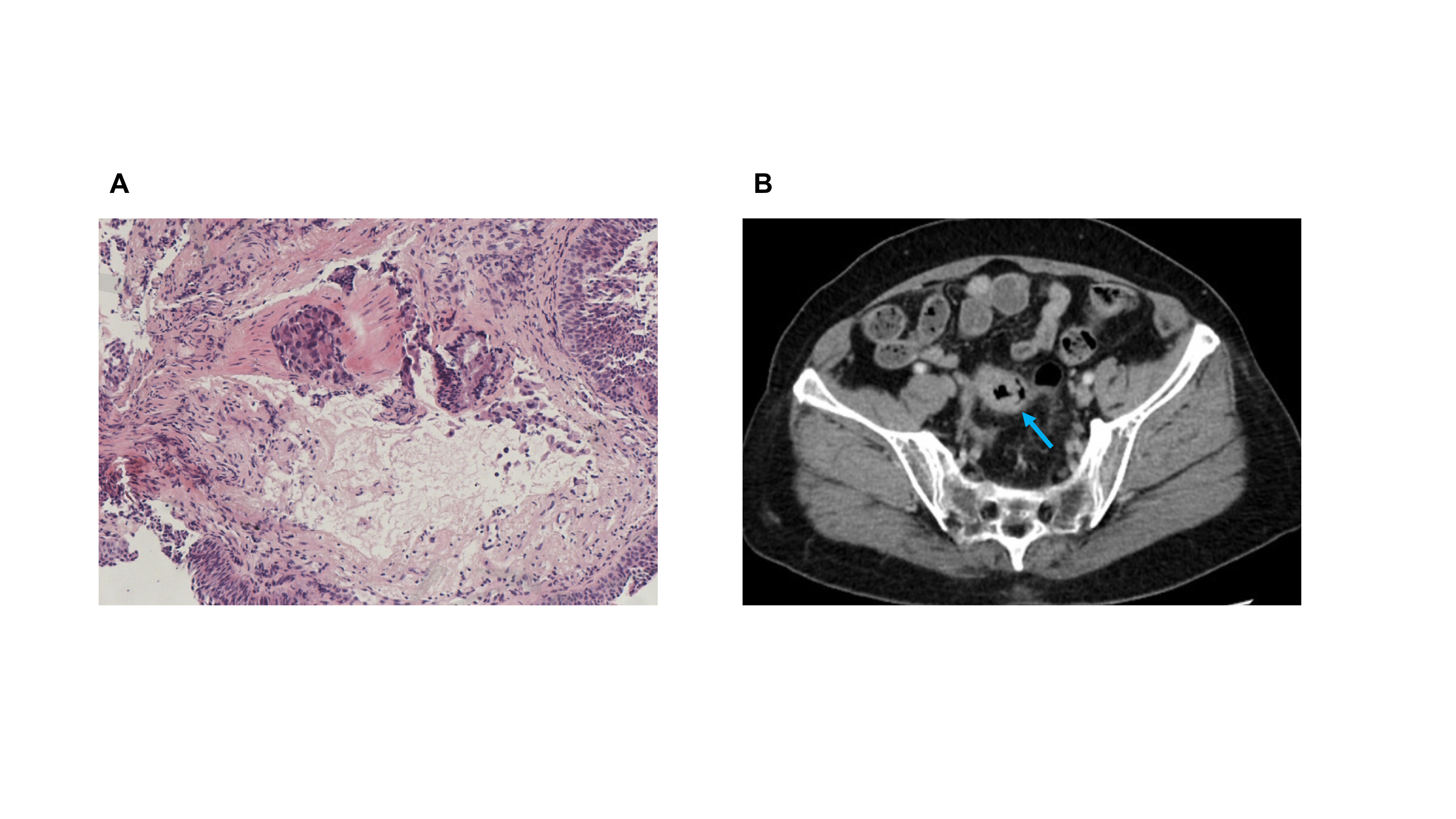

Supplement: Supplementary file 1 — Fig. S1 (A) Pathology of lung cancer. (B) Abdominal CT of colorectal cancer. [file TCA-13-1076-s001.tif]
